# *Aloe vera* Gel Drying by Refractance Window^®^: Drying Kinetics and High-Quality Retention

**DOI:** 10.3390/foods10071445

**Published:** 2021-06-22

**Authors:** Alfredo A. Ayala-Aponte, José D. Cárdenas-Nieto, Diego F. Tirado

**Affiliations:** 1 School of Food Engineering, Universidad del Valle, Cali 760031, Colombia; alfredo.ayala@correounivalle.edu.co (A.A.A.-A.); jodacani@gmail.com (J.D.C.-N.); 2 Grupo de Investigación en Innovación y Desarrollo Agropecuario y Agroindustrial (IDAA), Campus Piedra de Bolívar, Universidad de Cartagena, Cartagena de Indias 130015, Colombia

**Keywords:** fourth generation drying technology, color change, rehydration, vitamin C, vitamin E

## Abstract

In most cases, conventional drying produces inferior quality products and requires higher drying times. A continuous pilot Refractance Window^®^ equipment was used to dry *Aloe vera* gel slabs of 5 and 10 mm thick at 60, 70, 80, and 90 °C, seeking a dry product with high-quality retention. Based on five empirical models, drying kinetics, diffusion coefficient, and activation energy were analyzed. Midilli–Kuck was the best predicting model. Short drying times (55–270 min) were needed to reach 0.10 g water/g solid. In addition, the technique yielded samples with high rehydration capacity (24–29 g water/g solid); high retention of color (∆*E*, 3.74–4.39); relatively low losses of vitamin C (37–59%) and vitamin E (28–37%). Regardless of the condition of temperature and sample thickness, a high-quality dried *Aloe vera* gel could be obtained. Compared with other methods, Refractance Window^®^ drying of *Aloe vera* achieved shorter drying times with higher quality retention in terms of color, vitamins C and E, and rehydration. Finally, the dried *Aloe vera* gel could be reconstituted to a gel close to its fresh state by rehydration.

## 1. Introduction

The *Aloe vera* plant is native to Africa, although it is cultivated worldwide in most tropical and subtropical regions due to its ability to adapt to different climates [1]. Leaves of the aloe plant are made of three parts: an odorless and clear inner gel, a bitter yellowish sap known as latex, and a thin outer layer rind. The clear inner gel contains most of the bioactive components such as flavonoids, terpenoids, lectins, fatty acids, anthraquinones, mono- and polysaccharides (pectins, hemicelluloses, glucomannan), tannins, sterols (campesterol, *β*-sitosterol), enzymes, salicylic acid, minerals (calcium, chromium, copper, iron, magnesium, manganese, potassium, phosphorus, sodium, and zinc), and vitamins (A, C, E, *β*-carotene, B1, B2, B3, B6, choline, B12, folic acid) [2]. Additionally, this gel has moisture ranging from 99.0 to 99.5% (wet basis, *wb*) and 0.5% *wb* to 1.0% *wb* dry matter [3].

The aloe gel has been the subject of much scientific study over the last few years regarding several claimed therapeutic properties: immunomodulatory, wound and burn healing, hypoglycemic, anticancer, gastro-protective, antifungal, and anti-inflammatory properties [4]. Consequently, *A. vera* gel has been an important ingredient in food products as a source of bioactive components [5]. The global *A. vera* market was worth USD 602 million in 2019, and it is expected to reach USD 915 million by 2025, growing at a compound annual growth rate (CAGR) of 8.60% from 2020 to 2025. The main reason for this growth may be related to rising health-consciousness, since consumers now prefer products containing natural ingredients (such as aloe gel), which are perceived to be safer and healthier than their processed or chemical counterparts [6].

The *A. vera* gel is highly perishable after harvesting due to its high content of water, which makes it susceptible to microbiological damage. Because of this, the potential use of aloe gel must involve some kind of processing to conserve the bioactive components, since it is necessary to preserve the physicochemical and nutritional properties and, at the same time, increase the shelf life of the gel [7,8]. Regarding this, *A. vera* gel has been dried by several methods for preservation, such as hot air drying [9,10,11], spray drying [12], osmotic dehydration [13], and combined methods such as hot air in combination with far-infrared radiation and high-voltage electric field [14]. When drying foods, a very important factor is to achieve good quality indicators such as color, flavor, texture, nutrient availability, among others; all of which determine the food’s value and consumer acceptance on dehydrated food products [7]. However, in most cases, these conventional drying methods produce inferior quality products and require higher drying times [7,15,16].

Drying is a highly energy-intensive unit operation due to the latent heat of evaporation required, accounting for roughly 20% of the total energy consumed in the food industry. Over 85% of industrial food dryers are convective, with mostly hot air as the media for heat transfer; often resulting in significant levels of changes in product quality from the initial fresh-like form [17]. Along with food quality retention, energy efficiency is equally important in the drying process, which is why both aspects are the subject of intensive research and development on food science [18,19,20,21]. Among the technologies currently deployed for the drying of valuable products, freeze-drying is very expensive, and despite the high-quality of its products, its use is limited to high-value products [15,18,22]. Thus, there is a strong need for developing alternative drying technologies considering operational capacity, process control, time requirements, cost economics, product quality, safety, and environmental aspects. In this regard, Refractance Window^®^ (RW) is an emerging drying technique with a potentially positive impact on the food industry in terms of scalability, energy efficiency, cost, and end-product quality [8,23].

RW drying is one such novel fourth generation drying technology, which has become attractive for applications in the food industry especially because it offers dried products of high quality combined with lesser drying time requirements [23]. This technique is relatively inexpensive, environmentally friendly, and can be applied to various foods in pureed/juiced or diced forms, which grants versatility to the technique. It uses water as a heating medium at a temperature below boiling point, and the product is placed on a Mylar plastic film that is relatively transparent to infrared radiation from water [24]. During RW drying, all three modes of heat transfer are involved: convection, conduction, and radiation. The combination of these mechanisms allows the RW process to be short, ensuring high retention of product quality [7,8,25].

The objective of this work was to study the drying of *A. vera* gel using the RW technique, by varying the operating temperature and sample thickness. Drying kinetics and diffusion coefficients were obtained and analyzed. Additionally, physicochemical (color, volume, and rehydration) and nutritional (vitamins C and E) characteristics were evaluated to assess if the RW process would yield high-quality dried end-products. To the best of our knowledge, there is no unified analysis of all these aspects together in the literature.

## 2. Materials and Methods

### 2.1. Raw Material

*A. vera* leaves were purchased from a supermarket in Cali (Colombia). The leaves were stored at 4 °C until use. They were selected for their color, size, and consistency. The leaves were washed with potable water; the outer layer rind was removed from the gel; then, slabs 25 mm long, 15 mm wide, and of two thicknesses (5 mm and 10 mm) were prepared. A slab geometry was selected, as it is an easy way to cut the sample at an industrial level. In addition, this was the geometry analyzed for the mathematical modeling of the drying kinetics.

### 2.2. Chemicals and Reagents

Monobasic potassium phosphate (KH_2_PO_4_, ≥99.0%), acetic acid (glacial, ≥99.7%), ascorbic acid (99.0%), vitamin E (α-tocopherol, ≥96.0%), methanol (≥99.9%), isopropanol (99.9%), and chloroform (99.9%) were all provided by Sigma-Aldrich (Colombia).

### 2.3. Refractance Window^®^ Drying

**Figure 1:**
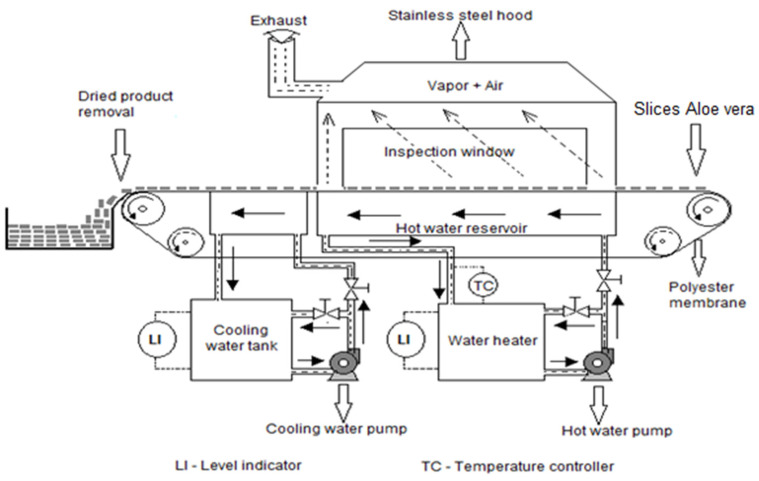
Schematic diagram of the Refractance Window^®^ continuous pilot dryer used in this work. Adapted from Ocoró-Zamora and Ayala-Aponte [27].

The *A. vera* gel samples were dried in a piece of continuous pilot equipment (CEI-ROBOTS, Colombia) to reproduce the RW principle. The apparatus consisted of a tank (0.6 × 0.4 m) filled with water, on the surface of which was a 0.25 mm Mylar film transparent to infrared energy. The water heating temperatures were 60, 70, 80, and 90 °C. The water was recirculated at a rate of 2.74 L/min. The slices to be dried were placed spaced 20 mm apart on the Mylar membrane and collected at different times to build the drying curve using a flat metal spatula. The air velocity of the exhaust fan from the stainless-steel hood was 50 mm/min. In this work, the moisture content was determined in an oven (Digiheat, J.P. Selecta, S.A., Colombia) at 103 °C until the weight did not vary more than 0.1%, according to the method 925.10 from AOAC [26]. A schematic representation of the pilot equipment is shown in Figure 1.

### 2.4. Mathematical Modeling of Drying Kinetics

Fick’s second law was used for the mathematical modeling of the drying kinetics, using the moisture ratio (MR) as the dependent variable described in Equation (1):

(1) $$MR=\frac{X_{t}-X_{e}}{X_{0}-X_{e}}$$

where *X_t_*, *X*_0_, and *X_e_* are time *t*, initial, and equilibrium moisture content (dry basis, *db*). Considering that the RW equipment employed in this study (see Figure 1) does not control the relative humidity of the air in contact with the material and that *X_e_* is relatively small compared to *X_t_* and *X*_0_, Equation (1) was simplified to Equation (2):

(2) $$MR=\frac{X_{t}}{X_{0}}$$

According to the time frame of the process (*t*) and considering the geometry of a semi-infinite slab with *L* thickness, the first term of the series was developed as Equation (3), where *i* is a positive integer (*i* = 1, 2, 3…), thus obtaining Equation (4). The effective moisture diffusivity (*D_eff_*) value was calculated by plotting *lnMR* versus drying time from the experimental data. The plot resulted in a straight line with a negative slope (*K*) that was related to *D_eff_* by Equation (5). The Arrhenius relation was used to evaluate the dependence of *D_eff_* as a function of temperature (*T*), determining the activation energy (*E_a_*) and the Arrhenius factor (*D*_0_), as shown in Equation (6); where *R* was the gas constant (8.314 × 10^−3^ kJ/mol):

(3) $$MR=\overset{\infty }{\underset{i=0}{\sum }}\frac{8}{{\left(2i+1\right)}^{2}\pi^{2}}\cdot \exp\left(\frac{-D_{eff}\left(2i+1\right)\pi^{2}t}{4L^{2}}\right)$$

(4) $$MR=\frac{8}{\pi^{2}}\exp\left(\frac{-D_{eff}\pi^{2}t}{4L^{2}}\right)$$

(5) $$K=\frac{D_{eff}\pi^{2}}{4L^{2}}$$

(6) $$D_{eff}=D_{0}\exp\left(-\frac{E_{a}}{RT}\right)$$

Experimental moisture values during *A. vera* gel drying were fitted to five (5) empirical kinetic models widely used in food drying and shown in Table 1.

The goodness of fit of the proposed models was assessed through the correlation coefficient (*R*^2^) with Equation (7); the sum of squares due to error (*SSE*) with Equation (8); the root mean square value (*RMSE*) with Equation (9); Chi-square (*X*^2^) with Equation (10):

(7) $$R^{2}=1-\frac{\sum_{i=1}^{N}{\left(MR_{cal}-MR\right)}^{2}}{\sum_{i=1}^{N}{\left(\bar{MR_{cal}}-MR_{exp}\right)}^{2}}$$

(8) $$SSE=\frac{1}{N}\overset{N}{\underset{i=1}{\sum }}{\left(MR_{exp}-MR_{cal}\right)}^{2}$$

(9) $$RMSE={\left[\frac{1}{N}\overset{N}{\underset{i=1}{\sum }}{\left(MR_{cal}-MR_{exp}\right)}^{2}\right]}^{1/2}$$

(10) $$X^{2}=\frac{\sum_{i=1}^{N}\left(MR_{cal}-MR_{exp}\right)}{N-Z}$$

where *N* is the number of observations; *Z* is the number of model parameters; *MR_cal_* and *MR_exp_* are the calculated and experimental *MR*, respectively.

### 2.5. Volume Change

The volume of the *A. vera* gel slabs were measured by using a digital calibrator (Red Line, Shelby County, IA, USA) with an accuracy of ±0.01 mm. The initial and final length, width, and thickness of each sample were measured three times at different points, obtaining averages for each value, to calculate the initial volume (*V*_0_) and volume at each drying time (*V_t_*).

### 2.6. Color

The color of samples was measured by a spectrophotometer (Hunter Lab ColorFlex, United States), which was used to obtain the coordinates luminosity (L*), green–red (a*), and blue–yellow (b*), using D65 as illuminant reference and the observer 10°. From the L*, a*, and b* coordinates, the total color variation (∆*E*) in each treatment was calculated using Equation (11), where the subscripts *0* and *t* refer to initial and time measurements, respectively.

(11) $$∆E=\sqrt{{\left(L_{0}^{*}-L_{t}^{*}\right)}^{2}+{\left(b_{0}^{*}-b_{t}^{*}\right)}^{2}+{\left(a_{0}^{*}-a_{t}^{*}\right)}^{2}}$$

### 2.7. Rehydration

Rehydration kinetics were carried out at 25 °C with initial moisture content in samples of 0.10 g water/g solid. Dried samples were placed in containers with distilled water, with a 1:40 solid to liquid ratio. Samples were removed at different times up to 360 min. During the first hour, samples were removed at 5, 20, and 60 min; then, every 30 min up to 120 min; finally, every 60 min until 360 min.

### 2.8. Vitamins

The measurement of vitamins C and E were performed for dry samples with an approximate moisture content of 0.10 g water/g solid.

#### 2.8.1. Vitamin C

Vitamin C was determined by using the United States Pharmacopoeia methodology [28]. A Shimadzu LC-2010A (Shimadzu Corporation, Kyoto, Japan) high-performance liquid chromatograph (HPLC) was used, which was programmed at a wavelength of 244 nm, flow rate 1 mL 0.2 M KH_2_PO_4_/min (pH 2.5) as mobile phase, injection volume of 20 µL, and run time of 15 min. Two grams of sample was weighed and diluted to a volume of 20 mL with 0.48% *v*/*v* acetic acid. Subsequently, it was taken to an ultrasonic cleaner (ROVSUN, Colombia) for 15 min. The samples were filtered through 0.45 μm PVDF membrane and deposited in 2 mL amber vials. The ascorbic acid standard that was used to quantify the samples was 5.04 mg/100 g solid. The sample peaks were identified according to the retention time and absorbance spectra compared to the standards. The minimum detection level of the HPLC chromatograph was 0.04 mg/100 g solid.

#### 2.8.2. Vitamin E

A Shimadzu LC-2010A HPLC was used at a wavelength of 280 nm, flow rate 0.8 mL isopropanol: methanol (50:50 *v*/*v*)/min, injection volume of 50 μL, and 20 min run time. Two grams of sample was weighed and diluted to a volume of 20 mL with chloroform: methanol (50:50 *v*/*v*). It was then taken to ultrasound for 15 min. Samples were filtered through 0.45 μm PVDF membrane and deposited in 2 mL amber vials. The standard vitamin E concentration was 0.77 mg/100 g solid. Sample peaks were identified according to retention time and absorbance spectra compared to standards. The minimum detection level of the HPLC chromatograph was 0.05 mg/100 g solid.

### 2.9. Experimental Design and Statistical Analysis

The experiments were conducted following a multilevel factorial design with two factors: *A. vera* gel slabs thickness (5 and 10 mm) and water heating temperatures (60, 70, 80, and 90 °C). The experiments were performed in triplicate, and the analyses were repeated at least twice (*n* = 2 × 3). Mean and standard deviation were calculated for all data. Response variables (moisture content, MR, diffusivity coefficient, activation energy, volume change, color change, rehydration, vitamin C loss, and vitamin E loss) were submitted to analysis of variance (ANOVA). When statistical differences at a significance level of 5% (*p* < 0.05) were found, Tukey’s analysis was performed to determine the influence of the selected factors. Data processing was carried out using Minitab 18 statistical program (Minitab, State College, PA, USA). Likewise, non-linear regression of the mathematical models was performed using SPSS 23 software (IBM, Armonk, NY, USA).

## 3. Results and Discussion

### 3.1. Drying

**Figure 2:**
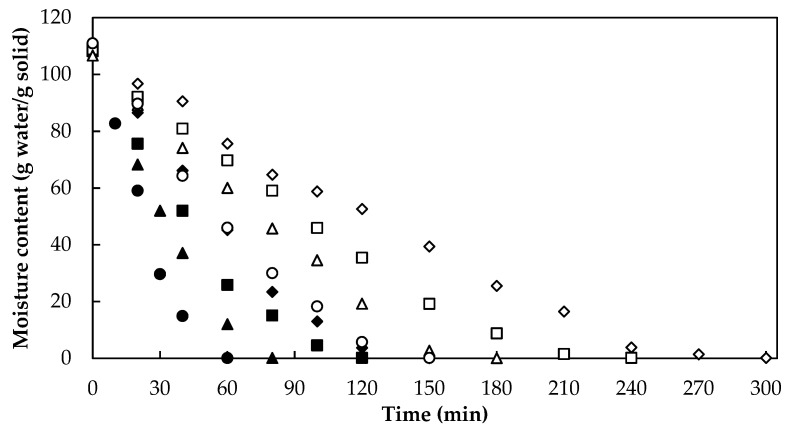
Drying curve of *Aloe vera* gel slabs with 5 mm (60 °C, ♦; 70 °C, ■; 80 °C, ▲; 90 °C, ●) and 10 mm (60 °C, ◊; 70 °C, □; 80 °C, ∆; 90 °C, ○) thickness.

Figure 2 shows the drying curves of *A. vera* samples obtained by RW. The moisture content of fresh *A. vera* samples was (110 ± 1) g water/g solid. According to the ANOVA analysis, drying temperature and sample thickness showed a significant effect (*p* < 0.05) on moisture content in *A. vera* gel slabs dried by RW. It can be seen in Figure 2 that the higher the temperature and the lower the thickness, the shorter the processing time is to reach the same moisture level. Similar behavior in the effect of temperature was observed during the drying of *A. vera* gel by other authors using hot air drying [10,29], vacuum drying [30], and osmotic dehydration [13]. Regarding the effect of thickness on drying time, similar behavior was observed during RW drying of mango slices [24], papaya puree [27], and kiwi slices [31].

Figure 2 shows that to obtain an approximate final moisture content of 0.10 g water/g solid, *A. vera* gel slabs with 5 mm thickness needed 145, 120, 81, and 55 min at 60, 70, 80, and 90 °C, respectively; while those samples with 10 mm thickness had longer drying times: 270 min at 60 °C, 220 min at 70 °C, 160 min at 80 °C, and 145 min at 90 °C, respectively. It is worth noting that these RW drying times were lower than those reported in other works involving *A. vera* gel drying by hot air convective drying method under relatively similar conditions: 210–800 min at 50–90 °C by Vega et al. [10]; 600 min at 60 °C by Miranda et al. [9]; 150–300 min at 50–80 °C by Sabat et al. [29]. Finally, Sriariyakul et al. [14] required times longer than 360 min during the drying of *A. vera* puree with hot air in combination with far-infrared radiation and a high-voltage electric field.

The rapid loss of water by RW was the result of the high mass and energy transfer that occurred in aloe slabs, since during RW drying, all three modes of heat transfer are active. Although in typical cases, conduction heat transfer predominates, the relative role of each mode of heat transfer depends on the water resistance offered by each transfer mode. While conduction, convection, and radiation occur at the hot water–film interface, conduction and radiation occur through the film, and convection occurs at the air–film interface [8].

The reduction in drying time due to the effect of temperature might be explained by the higher temperature gradient between the heat source (water) and the gel slabs, which favored a rapid removal of water from the sample due to the increase in heat transfer [23]. According to the works of Jha et al. [30] and Beigi [32], the increase in temperature increases heat transfer, evaporation rate, and water migration from inside to the sample surface. On the other hand, similar to most techniques, products being dried first go through a constant drying rate period that is heavily dependent on product thickness. The absorption of transmitted radiation by the product is governed by Beer’s law and higher product thickness implies lesser effects due to infrared radiation [8]. With thinner slabs, the sample heats up faster; thereby allowing water molecules to escape to the surface of the sample in a shorter time, along with the fact that water needs to move a shorter distance. Therefore, an increase in product thickness generated a decrease in mass flow, thus a lower drying rate [8,23].

Energy requirements of any unit operation are crucial in the industry perspective. In this regard, the thermal efficiency of an RW drying system is usually in the range of 52–77%, which are values comparable to drum drying [23]. RW has a comparable operational capacity with spray dryers and drum dryers, though these capacities are much lower than rotary dryers [8,33]. Compared to spray drying, RW energy requirement is 33% lesser. Likewise, hot air drying systems offer only 50% efficiency of RW dryers [8]. Finally, the cost of an RW equipment is approximately one-third to one-half of that of a freeze dryer to dry a similar amount of product, while the energy costs to operate RW dryers are less than half of freeze dryers [8,23]. This is an important consideration, since RW has demonstrated the capability to produce dried products of similar quality to those obtained by freeze-drying [34].

The main factors to be taken into account for the preservation of the functional properties of *A. vera* gel are temperature and exposure time, since the longer the exposure time, the more degradation of nutrients occurs [3,5]. Artunduaga-Antury et al. [35] claimed that the drying techniques that best preserve the functional properties of *A. vera* gel are freeze-drying and RW, since both allow the use of low temperatures and short drying times. These authors also stated that both techniques avoid cross-contamination, preserve physical properties (color), and active components such as vitamins and antioxidants. Other authors have claimed the similarities between dried products obtained by freeze-drying and RW [22,23]. Though, the RW drying systems are simple and relatively inexpensive when compared with freeze-drying, which usually needs large installations to be economical [23]. Hence, according to the savings in energy, from the commercial point of view, the RW technique might be a low-cost scalable option for the continuous production of high-quality dried products [8,23].

### 3.2. Effective Diffusivity Coefficient and Activation Energy

Table 2 shows the values of the average effective diffusivity coefficient (*D_eff_*) and activation energy (*E_a_*) for *A. vera* gel samples with 5 and 10 mm thicknesses dried by RW at different temperatures.

ANOVA showed that there was a significant effect (*p* < 0.05) of temperature and sample thickness on *D_eff_*, which increased with increasing temperature and sample thickness. It can be noted in Table 2 that the *D_eff_* for aloe gel slabs with 5 mm thickness ranged from 7 × 10^−^⁹ to 19 × 10^−^⁹ m^2^/s; while for 10 mm thickness, it was between 13 × 10^−^⁹ to 27 × 10^−^⁹ m^2^/s. These *D_eff_* values were within the order of 10^−9^ m^2^/s for *A. vera* drying [29].

The increase in *D_eff_* with increasing temperature in both sample thicknesses could be associated with the increase in heating energy and activity of water molecules, allowing greater moisture diffusion [36]. An increase in temperature decreases the viscosity of water and, subsequently, the fluid outflow resistance. This phenomenon leads to the diffusion of water molecules in the capillaries of the product, increasing the moisture diffusivity value [29]. This same behavior was observed during the drying of *A. vera* by hot air drying [10], osmotic dehydration [13], and vacuum drying [30]. In addition, similar behavior was observed during the drying of other foods such as banana slices [37] and *Butia capitata* [38].

Regarding the increase in *D_eff_* with increasing thickness, this could be due to different factors. On the one hand, the diffusion model used assumed only one direction of water diffusion: from the interior to the surface of the sample. Since lateral diffusion was negligible, diffusion along the thickness of the sample denoted higher water migration for samples with a thickness of 10 mm [39]. On the other hand, drying at high temperatures produced a shrinkage phenomenon on the surface of the sample, being of greater effect on the thinner samples. The rapid initial evaporation rate and migration of soluble solids to the surface could have caused a decrease in the drying rate, thus reducing water diffusion [39,40]. Similar results were found by other researchers on different foods: banana slices [39], papaya puree [27], and tomato slices [41].

The activation energy (*E_a_*) is the energy required by the water molecules before starting their diffusion into the food during drying. High *E_a_* values are associated with a stronger binding of water to the feed structure. Table 2 shows *E_a_* of 5 and 10 mm thick *A. vera* gel samples. In this study, *E_a_* was higher at a lower thickness (5 mm) with a value of 33 kJ/mol; while for the 10 mm sample thickness, it was 26 kJ/mol. Similar behavior was reported by Nguyen and Price [39] during the drying of banana slices, with *E_a_* values of 40 and 35 kJ/mol for thicknesses of 10 and 20 mm, respectively. Thus far, no clear relationship between *E_a_* and sample thickness can be established, since the increase in thickness does not always imply a decrease in *E_a_* [41]. The *E_a_* values in *A. vera* gel slabs from this work were within the values found during drying of *A. vera* [10,29,30] and banana [37,42].

### 3.3. Drying Kinetic Models

Table 3 shows the kinetic parameters of five models (see Table 1), along with the quality criteria for the evaluation of the fit: *R*^2^, *χ*^2^, *RMSE*, and *SSE*. All models presented a good fit; though the Midilli–Kuck model was the one that best fitted the experimental values in all drying conditions, since it had *R*^2^ values closer to 1.0 and *RMSE* and *χ*^2^ closer to zero (see Table 3).

Table 3 also shows that the parameter *k* increased with increasing temperature and decreased with sample thickness. This indicated the dependence of temperature on *k* for thicknesses of 5 and 10 mm. Furthermore, *k* could be associated with the ease of water removal from the sample, considering this parameter as a pseudo-diffusivity [10,37]. In contrast, the parameters *n*, *a*, and *b* did not present any behavior associated with temperature and/or sample thickness. Similar results were reported during the drying of *A. vera* [10,30] and tomato slices [41].

**Figure 3:**
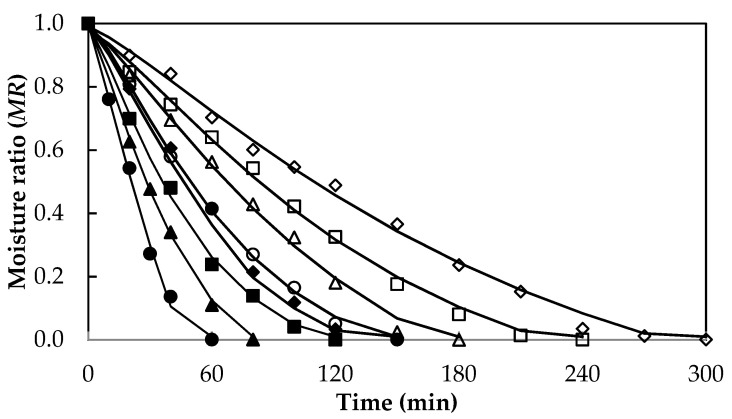
Drying kinetics adjusted by Midilli–Kuck model for *Aloe vera* gel slabs with 5 mm (60 °C, ♦; 70 °C, ■; 80 °C, ▲; 90 °C, ●) and 10 mm (60 °C, ◊; 70 °C, □; 80 °C, ∆; 90 °C, ○) thickness.

The Midilli–Kuck model successfully represents the drying kinetics in different foods, as reported by other authors during the drying of papaya by RW [27]; lemon slices by oven drying [43]; persimmon by osmo-convective drying [44]; banana slices by hot air drying [37]. Figure 3 shows the experimental drying curves and those fitted by the Midilli–Kuck model for samples with 5 and 10 mm thicknesses from this work.

### 3.4. Volume Change

**Figure 4:**
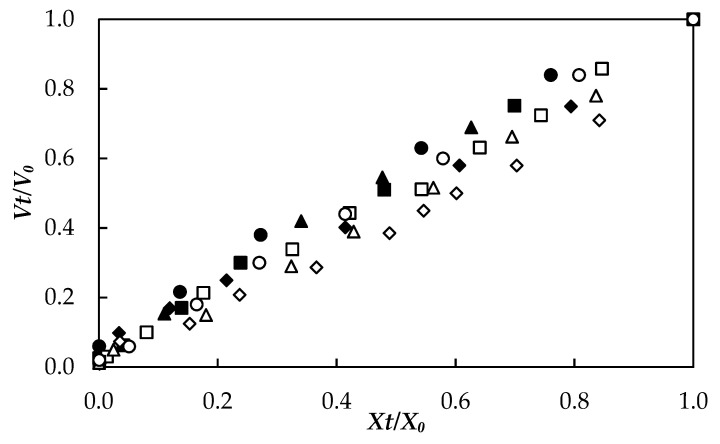
Relationship between volume change and moisture content during drying of Aloe vera gel slabs with 5 mm (60 °C, ♦; 70 °C, ■; 80 °C, ▲; 90 °C, ●) and 10 mm (60 °C, ◊; 70 °C, □; 80 °C, ∆; 90 °C, ○) thickness.

The relationship between the volume change factor (*V_t_*/*V*_0_) and humidity (*X_t_*/*X*_0_) of dry *A. vera* slabs with thicknesses of 5 and 10 mm are shown in Figure 4. The error bars are not presented to make Figure 4 easier to see, yet the standard deviations ranged from 0.01 to 0.05. It is observed in Figure 4 that the volume change factor decreases (shrinkage increases) linearly with decreasing moisture content. Similar results have been obtained during the drying of mango by RW [24] and potato by thin-layer drying [45]. ANOVA showed a significant effect (*p* < 0.05) of the temperature and thickness factors on *A. vera* gel volume loss. Less shrinkage or less volume change was found in the samples with increasing drying temperature. Such behavior may be attributed to the lower moisture content of the outer surface, which could induce a rubbery-glass transition with the possible formation of a rigid crust on the outer layer that helps to fix the sample size [46].

Regarding thickness (see Figure 4), lower shrinkage values (higher volume change factor) were observed in the thinner samples (5 mm), possibly due to the shorter time required to dry these slabs; while, the 10 mm thick samples required longer drying times, which could cause greater changes in their structure associated with cellular shrinkage. According to Mayor and Sereno [46], during drying, tensions are generated in the cellular structure that with prolonged drying times can cause a structural collapse in the pores and compartments of the cellular tissue of the dehydrated material.

### 3.5. Color Change

**Figure 5:**
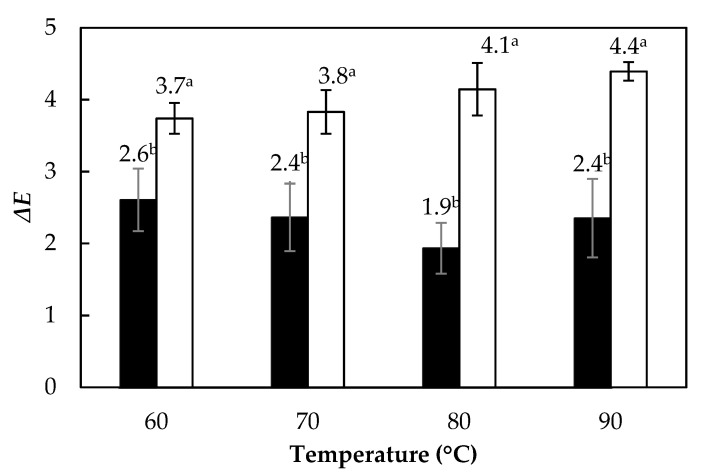
Total color change (∆*E*) during drying of *Aloe vera* gel slabs with 5 mm (■) and 10 mm (□) thickness. Values with a different letter (a, b) are significantly different (*p* < 0.05).

Figure 5 shows the total color change (∆*E*) of *A. vera* gel samples dried to a moisture content of 0.10 g water/g solid. The drying temperature in both thicknesses did not present significant differences (*p* > 0.05) in the values of L*, a*, and b* (values not shown). Consequently, there was no significant difference in the ∆*E* in the aloe gel. On the other hand, as observed in Figure 5, the thickness had a significant influence (*p* < 0.05) on ∆*E*, showing higher values in the 10 mm thick samples. The aforementioned was attributed to the longer time required to dry the 10 mm thin samples, which took twice the time of thinner samples (5 mm) to reach the same moisture content. However, these total color changes are considered low, varying between 3.74 and 4.39 units. Thus, when visually observing the dehydrated aloe gels, no color differences were observed concerning the fresh samples. Our ∆*E* values were lower than those reported by Miranda et al. [9] during drying of *A. vera* with hot air. These authors found Δ*E* values between 15 and 22 units employing temperatures between 50 and 90 °C and using 10 mm thin aloe gel slabs. Our results proved the feasibility of the RW method to preserve the natural color of the samples to be dried.

### 3.6. Rehydration

The relevance of studying the rehydration process in dry foods lies in the need to restore the initial properties of the fresh food before its consumption, trying to recover as much as possible its original shape, size, and moisture. The rehydration rate is one of the main parameters to determine the quality of the dried product, which, after being rehydrated, could be used in different formats, e.g., soups. Thus, for example, dehydrated *A. vera* used as an ingredient in instant soups and sauces in flaked or powdered form are typical examples of the rehydration of dried vegetables before consumption. Moreover, rehydration can be considered as a measure of the damage to the material caused by the drying process. This shows the need for a better understanding of the rehydration process in dried foods [47].

**Figure 6:**
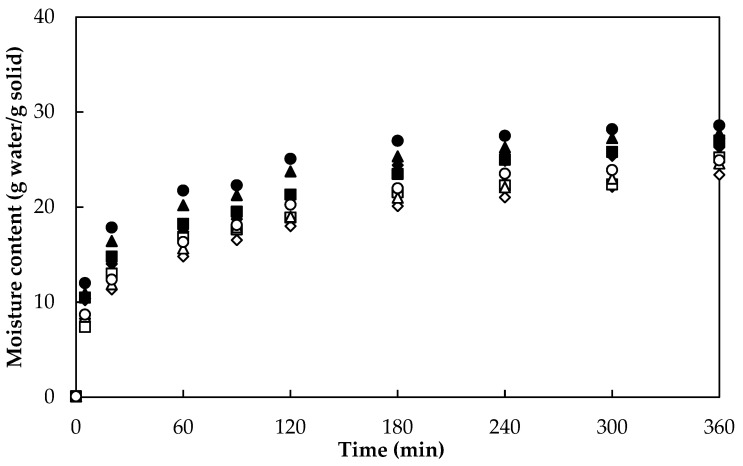
Rehydration curve of *Aloe vera* gel slabs with 5 mm (60 °C, ♦; 70 °C, ■; 80 °C, ▲; 90 °C, ●) and 10 mm (60 °C, ◊; 70 °C, □; 80 °C, ∆; 90 °C, ○) thickness at 25 °C.

Rehydration is not the reverse of drying, since water that is removed from food during dehydration cannot be replaced in the same way when the food is rehydrated. Rehydration of a food sample involves three simultaneous processes: (i) first, the imbibition of water into the dried material; (ii) then swelling; finally, (iii) leaching of soluble materials. The rate and extent of rehydration depend on the extent of disruption to the cellular structure and chemical changes caused by dehydration. Figure 6 shows the rehydration curves of *A. vera* gel slabs dried at two levels of thickness (5 and 10 mm) and four levels of temperature (60, 70, 80, and 90 °C). ANOVA showed a significant effect (*p* < 0.05) of both sample thickness and drying temperature on the rehydration of aloe gel slabs. Figure 6 shows typical rehydration behavior in all treatments, with rapid water absorption in the first few minutes (i). Then, water absorption slows down (ii) to a constant rate or equilibrium after 360 min (iii). These three periods have been observed by other researchers in the rehydration of *A. vera* slabs [48,49], pumpkin slices [50], and brown seaweeds [51]. The initial rapid absorption of water could have been due to the filling of capillaries on the surface of the sample [49]. The decrease in the rehydration rate could have been associated with the decrease in free capillaries due to water filling of intracellular spaces.

The equilibrium moisture content in the hydrated samples ranged from 24 to 29 g water/g solid. These results suggested the high water absorption capacity of the dehydrated *A. vera* gel, since the moisture content in the fresh state was 111 g water/g solid. When comparing these results with those of Vega-Gálvez et al. [49] for the drying of *A. vera* gel with hot air at 60 °C and later rehydration, it was found that the aloe gel dried by RW had a higher water absorption capacity in a shorter process time, which may be evidence that the *A. vera* dried by RW exhibited less structural damage concerning hot air drying, since structural damage during product drying causes a loss of rehydration capacity [52].

Greater water gain was observed in the rehydration of *A. vera* with increasing drying temperature. This behavior may be associated with an increase in porosity, since as the drying temperature increases, the water in the product comes out faster at the beginning of drying, creating large spaces that allow for greater water penetration in the rehydration of the sample. Rehydration capacity depends largely on the nature of the food porosity; high porosity in a product indicates high rehydration capacity [46]. Similar behavior was reported by Miranda et al. [48] during *A. vera* drying and by Tepe and Tepe [53] during apple drying. Likewise, according to Aral and Beşe [54], the porosity of hawthorn fruits increased with increasing drying temperature, allowing greater water entry in the rehydration process.

Finally, regarding thickness, it was observed that the thinner samples (5 mm) rehydrated faster than the 10 mm samples, reaching higher water gain. This result could be associated with the fact that the thinner samples presented less shrinkage or loss of volume, possibly showing less structural damage, thus facilitating the entry of water. It could also be associated with the fact that decreasing the thickness of the samples decreased the internal resistance to water penetration during rehydration. Similar results were found by Xiao et al. [55] during the rehydration of American ginseng.

### 3.7. Vitamins

Fresh *A. vera* gel had (114.1 ± 1.6) mg/100 g solid and (0.7 ± 0.3) mg/100 g solid of vitamin C and E, respectively. Miranda et al. [9] reported a relatively similar value of vitamin C in fresh *A. vera* (122 mg water/100 g solid) and a lower value of vitamin E (0.2 mg/100 g solid). Saberian et al. [56], on the other hand, reported a lower value of vitamin C in fresh aloe gel (85 mg/100 g solid). Table 4 shows the vitamin C and E contents of dried aloe slabs. The losses of vitamin C and E content of *A. vera* gel dehydrated by RW were quantified for moisture content of 0.10 g water/100 g solid and can also be found in Table 4. ANOVA showed a significant effect (*p* < 0.05) of temperature and sample thickness on vitamin C loss. On the contrary, temperature and sample thickness did not have a significant effect (*p* > 0.05) on vitamin E losses.

As shown in Table 4, significant losses (*p* < 0.05) of vitamins C and E were observed between the fresh and dry samples, with higher losses in vitamin C, which varied between 41 and 59% for the 5 mm thickness and between 29% and 50% for 10 mm. In the case of vitamin E, losses varied between 33 and 37% for 5 mm and between 28 and 32% for 10 mm. The higher loss of vitamin C was associated with the fact that it is one of the most heat-sensitive nutrients, whereas vitamin E is a thermostable element. Vitamin C is an indicator of quality retention in dehydrated foods, since it shows significant losses during drying [57,58]. Although vitamin E is a thermostable nutrient, it is susceptible to oxidation during drying [59,60].

To the best of our knowledge, there is no report in the scientific literature of vitamin losses in freeze-dried aloe vera gel. However, the losses of vitamin C by RW reported in this work were significantly lower than those reported for other drying methods of *A. vera* and other foods. Miranda et al. [9] reported vitamin C losses between 79 and 87% in *A. vera* gel when temperatures were between 60 and 90 °C during tray drying. The authors explained that these losses were probably due to the high temperature and long drying times with hot air, reaching times of 810 min; while in this work with RW, shorter times were achieved, ranging from 55 to 240 min. Ouyang et al. [61] reported vitamin C losses from 49 to 88% during hot air drying of pumpkin. Kurozawa et al. [62] found vitamin C losses from 48 to 76% during hot air drying of papaya cubes at temperatures between 40 and 70 °C. Jha and Sit [63] claimed losses of vitamin C between 74 and 87% during hot air drying of *Terminalia chebula*. Finally, significant losses of vitamin C were also reported in other foods such as guava pulp [64] and doum fruits [65] due to drying.

According to the above, it is evident that the use of RW in this work led to lower vitamin C losses compared to tray or hot air drying in *A. vera* and other foods. This may be due to the short exposure time of the product during RW drying, which results in less oxidation of the *A. vera* gel.

## 4. Conclusions

Continuous Refractance Window^®^ pilot equipment demonstrated the potential to produce high-quality *Aloe vera* gel dried slabs in this work. Regardless of the condition of temperature and sample thickness, a high-quality dried *Aloe vera* gel could be obtained. Additionally, when comparing the results of this work with other methods used to dry *Aloe vera* gel and puree reported in the literature, it was observed that by using Refractance Window^®^ drying, significantly shorter drying times were obtained with higher quality retention in terms of color, vitamins C and E, and rehydration.

It is reported in the literature that Refractance Window^®^ can produce dried products of similar quality to those obtained by freeze-drying [34], but its cost is ~30–50% of freeze dryers, and it requires <50% energy to dry the same mass of the product [8]. Therefore, the Refractance Window^®^ is an emerging drying technique that might be a low-cost scalable option for continuous production of high-quality dried products, with a potentially positive impact on the food industry in terms of scalability, energy consumption, product quality, safety, environmental impact, cost, and productivity. Therefore, along with shifting consumer trends towards high-quality foods, Refractance Window^®^ will surely find its way to being among the most preferred drying techniques in the food industry.

Refractance Window^®^ has great potential to produce dry *Aloe vera* gel of high commercial quality relatively similar to fresh gel. Thanks to the versatility of the technology, dry gels could be produced in the form of slices or flakes and subsequently converted into powder, which could be used as ingredients in various food processes such as health drinks, fruit or vegetable salads, ice cream, and confectionery, among other food supplements. Additionally, the dried *Aloe vera* gel could be reconstituted to a gel close to its fresh state by rehydration.

## Figures and Tables

**Table 1:** Mathematical models applied to the drying curves in the study.

Model Name	Model
Page	$MR=\exp\left(-kt^{n}\right)$
Modified Page	$MR=\exp\left[{\left(-kt\right)}^{n}\right]$
Henderson and Pabis	MR=a×exp(−kt)
Logarithmic	MR=a×exp(−kt)+b
Midilli–Kuck	$MR=a\times \exp\left(-kt^{n}\right)+bt$

*MR*, moisture ratio; *t*, time (min); *k*, *n*, *a*, and *b* are model parameters.

**Table 2:** Diffusivity coefficient (*D_eff_*) and activation energy (*E_a_*) during *Aloe vera* drying.

Thickness (mm)	*D_eff_* × 10^−^⁹ m^2^/s				*E_a_* (kJ/mol)
	60 °C	70 °C	80 °C	90 °C	
5	7 ± 0 ^e^	9 ± 1 ^e^	13 ± 1 ^d^	19 ± 1 ^c^	33 ± 2 ^a^
10	13 ± 1 ^d^	17 ± 1 ^c^	24 ± 1 ^b^	27 ± 2 ^a^	26 ± 2 ^b^

Different lowercase letters (a, b, c, d, and e) within the same row represent statistically significant differences at 5% significance.

**Table 3:** Model parameters and statistical results for *Aloe vera* gel slabs drying in this work.

Model	Parameters and Statistical Test	5 mm				10 mm			
		60 °C	70 °C	80 °C	90 °C	60 °C	70 °C	80 °C	90 °C
Page	*k* × 10^−3^	2.788	4.884	5.376	6.839	0.700	1.167	1.286	2.753
	*n*	1.538	1.358	1.484	1.536	1.478	1.453	1.502	1.422
	*R* ^2^	0.946	0.995	0.994	0.997	0.992	0.993	0.993	0.998
	*χ* ^2^	0.011	0.001	0.001	0.001	0.001	0.001	0.002	0.000
	*RMSE*	0.094	0.029	0.031	0.130	0.035	0.032	0.036	0.106
	*SSE*	0.009	0.001	0.001	0.017	0.001	0.001	0.001	0.011
Modified Page	*k* × 10^−3^	16.353	21.338	27.737	38.931	7.337	9.577	11.909	15.813
	*n*	1.538	1.358	1.484	1.536	1.478	1.453	1.502	1.422
	*R* ^2^	0.998	0.998	0.997	0.997	0.992	0.993	0.993	0.998
	*χ* ^2^	0.001	0.000	0.001	0.001	0.001	0.001	0.002	0.000
	*RMSE*	0.021	0.020	0.022	0.130	0.035	0.032	0.036	0.106
	*SSE*	0.000	0.000	0.000	0.017	0.001	0.001	0.001	0.011
Henderson and Pabis	*k* × 10^−3^	18.126	23.139	29.319	41.602	8.068	10.396	12.969	17.273
	*a*	1.064	1.038	1.036	1.052	1.083	1.064	1.066	1.059
	*R* ^2^	0.982	0.988	0.982	0.973	0.973	0.977	0.976	0.987
	*χ* ^2^	0.004	0.002	0.004	0.007	0.005	0.004	0.005	0.003
	*RMSE*	0.059	0.044	0.054	0.117	0.065	0.061	0.065	0.111
	*SSE*	0.003	0.002	0.003	0.014	0.004	0.004	0.004	0.012
Logarithmic	*k* × 10^−3^	10.541	15.254	15.558	24.307	3.401	5.012	5.272	10.056
	*a*	1.352	1.227	1.433	1.368	1.662	1.506	1.718	1.347
	*b*	−0.324	−0.216	−0.428	−0.345	−0.645	−0.497	−0.707	−0.325
	*R* ^2^	0.997	0.998	0.999	0.994	0.997	0.998	0.998	0.999
	*χ* ^2^	0.001	0.001	0.000	0.003	0.001	0.001	0.001	0.000
	*RMSE*	0.028	0.018	0.011	0.120	0.022	0.020	0.019	0.105
	*SSE*	0.001	0.000	0.000	0.014	0.000	0.000	0.000	0.011
Midilli–Kuck	*k* × 10^−3^	4.707	8.143	10.111	11.291	1.652	2.030	2.911	4.455
	*n*	1.229	1.224	1.229	1.190	1.243	1.283	1.249	1.275
	*a*	0.997	0.997	0.999	1.000	0.991	0.979	0.990	1.000
	*b* × 10^5^	−0.002	−0.001	−0.001	−0.007	−0.001	−0.001	−0.001	−0.001
	*R* ^2^	0.997	0.999	1.000	0.998	0.998	0.998	0.998	1.000
	*χ* ^2^	0.002	0.000	0.000	0.002	0.001	0.000	0.001	0.000
	*RMSE*	0.033	0.014	0.009	0.119	0.021	0.018	0.019	0.108
	*SSE*	0.001	0.000	0.000	0.009	0.000	0.000	0.000	0.012

**Table 4:** Vitamin C and E content in dried *Aloe vera* slabs with 5 and 10 mm thickness.

T (°C)	Vitamin C Content (mg/100 g Solid)		Vitamin C Loss (%)		Vitamin E Content (mg/100 g Solid)		Vitamin E Loss (%)	
	5 mm	10 mm	5 mm	10 mm	5 mm	10 mm	5 mm	10 mm
60	68 ± 1 ^a^	81 ± 2 ^a^	41 ± 1 ^a^	29 ±1 ^a^	0.48 ± 0.03 ^a^	0.52 ± 0.02 ^a^	33 ± 0 ^a^	28 ± 0 ^a^
70	63 ± 1 ^b^	76 ±1 ^b^	44 ± 1 ^b^	37 ± 1 ^b^	0.47 ± 0.04 ^a^	0.51 ± 0.03 ^a^	35 ± 0 ^a^	29 ± 0 ^a^
80	53 ± 1 ^c^	68 ± 1 ^c^	54 ± 1 ^c^	41 ±1 ^c^	0.47 ± 0.02 ^a^	0.50 ± 0.02 ^a^	35 ± 0 ^a^	30 ± 0 ^a^
90	47 ± 1 ^d^	57 ± 1 ^d^	59 ± 1 ^d^	50 ±1 ^d^	0.46 ± 0.03 ^a^	0.49 ± 0.01 ^a^	37 ± 0 ^a^	32 ± 0 ^a^

Values with a different letter (a, b, c, d) are significantly different (*p* < 0.05).

## Data Availability

The data used to support the findings of this study are available from the corresponding author upon request.

## References

[1] Kumar R., Singh A.K., Gupta A., Bishayee A., Pandey A.K. (2019). Therapeutic potential of Aloe vera—A miracle gift of nature. Phytomedicine.

[2] Hęś M., Dziedzic K., Górecka D., Jędrusek-Golińska A., Gujska E. (2019). Aloe vera (L.) Webb: Natural Sources of Antioxidants—A Review. Plant Foods Hum. Nutr..

[3] Añibarro-Ortega M., Pinela J., Barros L., Ćirić A., Silva S.P., Coelho E., Mocan A., Calhelha R.C., Soković M., Coimbra M.A. (2019). Compositional Features and Bioactive Properties of Aloe vera Leaf (Fillet, Mucilage, and Rind) and Flower. Antioxidants.

[4] Maan A.A., Nazir A., Khan M.K.I., Ahmad T., Zia R., Murid M., Abrar M. (2018). The therapeutic properties and applications of Aloe vera: A review. J. Herb. Med..

[5] Ahlawat K.S., Khatkar B.S. (2011). Processing, food applications and safety of aloe vera products: A review. J. Food Sci. Technol..

[6] (2020). Research and Markets Aloe Vera Gel Market: Global Industry Trends, Share, Size, Growth, Opportunity and Forecast 2020–2025.

[7] Vega-Mercado H., Marcela Góngora-Nieto M., Barbosa-Cánovas G.V. (2001). Advances in dehydration of foods. J. Food Eng..

[8] Raghavi L.M., Moses J.A., Anandharamakrishnan C. (2018). Refractance window drying of foods: A review. J. Food Eng..

[9] Miranda M., Maureira H., Rodríguez K., Vega-Galvez A. (2009). Influence of temperature on the drying kinetics, physicochemical properties, and antioxidant capacity of Aloe Vera (Aloe Barbadensis Miller) gel. J. Food Eng..

[10] Vega A., Uribe E., Lemus R., Miranda M. (2007). Hot-air drying characteristics of Aloe vera (Aloe barbadensis Miller) and influence of temperature on kinetic parameters. LWT Food Sci. Technol..

[11] Gulia A., Sharma H.K., Sarkar B.C., Upadhyay A., Shitandi A. (2010). Changes in physico-chemical and functional properties during convective drying of aloe vera (Aloe barbadensis) leaves. Food Bioprod. Process..

[12] Cervantes-Martínez C.V., Medina-Torres L., González-Laredo R.F., Calderas F., Sanchez-Olivares G., Herrera-Valencia E.E., Infante J.G.A., Rocha-Guzman N.E., Rodríguez-Ramírez J. (2014). Study of spray drying of the Aloe vera mucilage (Aloe vera barbadensis Miller) as a function of its rheological properties. LWT Food Sci. Technol..

[13] Pisalkar P.S., Jain N.K., Pathare P.B., Murumkar R.P., Revaskar V.A. (2014). Osmotic dehydration of aloe vera cubes and selec-tion of suitable drying model. Int. Food Res. J..

[14] Sriariyakul W., Swasdisevi T., Devahastin S., Soponronnarit S. (2016). Drying of aloe vera puree using hot air in combination with far-infrared radiation and high-voltage electric field: Drying kinetics, energy consumption and product quality evaluation. Food Bioprod. Process..

[15] Bao H., Zhou J., Yu J., Wang S. (2021). Effect of Drying Methods on Properties of Potato Flour and Noodles Made with Potato Flour. Foods.

[16] Abbaspour-Gilandeh Y., Kaveh M., Fatemi H., Aziz M. (2021). Combined Hot Air, Microwave, and Infrared Drying of Hawthorn Fruit: Effects of Ultrasonic Pretreatment on Drying Time, Energy, Qualitative, and Bioactive Compounds’ Properties. Foods.

[17] Ladha-Sabur A., Bakalis S., Fryer P.J., Lopez-Quiroga E. (2019). Mapping energy consumption in food manufacturing. Trends Food Sci. Technol..

[18] Nallan Chakravartula S., Moscetti R., Farinon B., Vinciguerra V., Merendino N., Bedini G., Neri L., Pittia P., Massantini R. (2021). Stinging Nettles as Potential Food Additive: Effect of Drying Processes on Quality Characteristics of Leaf Powders. Foods.

[19] Zhang W., Chen C., Pan Z., Zheng Z. (2021). Vacuum and Infrared-Assisted Hot Air Impingement Drying for Improving the Processing Performance and Quality of Poria cocos (Schw.) Wolf Cubes. Foods.

[20] Xia J., Guo Z., Fang S., Gu J., Liang X. (2021). Effect of Drying Methods on Volatile Compounds of Burdock (Arctium lappa L.) Root Tea as Revealed by Gas Chromatography Mass Spectrometry-Based Metabolomics. Foods.

[21] Ferreira J.P.D.L., Queiroz A.J.D.M., Figueirêdo R.M.F.D., Silva W.P.D., Gomes J.P., Santos D.D.C., Silva H.A., Rocha A.P.T., Paiva A.C.C.D., Chaves A.D.C.G. (2021). Utilization of Cumbeba (Tacinga inamoena) Residue: Drying Kinetics and Effect of Process Conditions on Antioxidant Bioactive Compounds. Foods.

[22] Minjares-Fuentes R., Femenia A., Comas-Serra F., Rosselló C., Rodríguez-González V.M., González-Laredo R.F., Gallegos-Infante J., Medina-Torres L. (2016). Effect of different drying procedures on physicochemical properties and flow behavior of Aloe vera (Aloe barbadensis Miller) gel. LWT.

[23] Nindo C.I., Tang J. (2007). Refractance Window Dehydration Technology: A Novel Contact Drying Method. Dry. Technol..

[24] Ochoa-Martínez C.I., Quintero P.T., Ayala A.A., Ortiz M.J. (2012). Drying characteristics of mango slices using the Refractance Window™ technique. J. Food Eng..

[25] Ortiz-Jerez M.J., Gulati T., Datta A.K., Ochoa-Martínez C.I. (2015). Quantitative understanding of Refractance Window™ drying. Food Bioprod. Process..

[26] Horwitz W., Latimer G., AOAC (2006). AOAC International Official Methods of Analysis.

[27] Ocoró-Zamora M.U., Ayala-Aponte A. (2013). Influence of thickness on the drying of papaya puree (Carica papaya L.) through Refractance WindowTM technology. Dyna.

[28] (2012). US Pharmacopeia USP-35/NF-30.

[29] Sabat M., Patel S., Kalne A.A. (2018). Influence of Temperature on Drying Kinetics of Aloe vera and Its Mathematical Modeling. Curr. J. Appl. Sci. Technol..

[30] Jha R.K., Prabhakar P.K., Srivastav P.P., Rao V.V. (2016). Influence of temperature on vacuum drying characteristics, functional properties and micro structure of Aloe vera (Aloe barbadensis Miller) gel. Res. Agric. Eng..

[31] Azizi D., Jafari S.M., Mirzaei H., Dehnad D. (2017). The Influence of Refractance Window Drying on Qualitative Properties of Kiwifruit Slices. Int. J. Food Eng..

[32] Beigi M. (2019). Drying of mint leaves: Influence of the process temperature on dehydration parameters, quality attributes, and energy consumption. J. Agric. Sci. Technol..

[33] Mahanti N.K., Chakraborty S.K., Sudhakar A., Verma D.K., Shankar S., Thakur M., Singh S., Tripathy S., Gupta A.K., Srivastav P.P. (2021). Refractance WindowTM-Drying vs. other drying methods and effect of different process parameters on quality of foods: A comprehensive review of trends and technological developments. Future Foods.

[34] Caparino O.A., Tang J., Nindo C.I., Sablani S.S., Powers J.R., Fellman J.K. (2012). Effect of drying methods on the physical properties and microstructures of mango (Philippine ‘Carabao’ var.) powder. J. Food Eng..

[35] Artunduaga-Antury K.L., Vargas-Rojas D.A., Barrera-Bermeo O.M. (2021). Conservation of aloe vera nutraceutical properties (Aloe barbadensis Miller) by drying techniques. Rev. Ing. Región.

[36] Xiao H.-W., Pang C.-L., Wang L.-H., Bai J.-W., Yang W.-X., Gao Z.-J. (2010). Drying kinetics and quality of Monukka seedless grapes dried in an air-impingement jet dryer. Biosyst. Eng..

[37] Macedo L.L., Vimercati W.C., Araújo C., Saraiva S.H., Teixeira L.J.Q. (2020). Effect of drying air temperature on drying kinetics and physicochemical characteristics of dried banana. J. Food Process. Eng..

[38] Macedo L.L., Corrêa J.L.G., Fonseca H.C., Araújo C.D.S., Vimercati W.C., Gandia R.M. (2020). Convective drying of Butia Capitata pulp: Effect of air temperature on kinetic and quality parameters. Res. Soc. Dev..

[39] Nguyen M.-H., Price W.E. (2007). Air-drying of banana: Influence of experimental parameters, slab thickness, banana maturity and harvesting season. J. Food Eng..

[40] Jafari S.-M., Azizi D., Mirzaei H., Dehnad D. (2016). Comparing Quality Characteristics of Oven-Dried and Refractance Window-Dried Kiwifruits. J. Food Process. Preserv..

[41] Sadin R., Chegini G.-R., Sadin H. (2014). The effect of temperature and slice thickness on drying kinetics tomato in the infrared dryer. Heat Mass Transf..

[42] Kumar P.S., Nambi E., Shiva K.N., Vaganan M.M., Ravi I., Jeyabaskaran K.J., Uma S. (2019). Thin layer drying kinetics of Banana var. Monthan (ABB): Influence of convective drying on nutritional quality, microstructure, thermal properties, color, and sensory characteristics. J. Food Process. Eng..

[43] Torki-Harchegani M., Ghasemi-Varnamkhasti M., Ghanbarian D., Sadeghi M., Tohidi M. (2016). Dehydration characteristics and mathematical modelling of lemon slices drying undergoing oven treatment. Heat Mass Transf..

[44] Sampaio R.M., Neto J.P.M., Perez V.H., Marcos S.K., Boizan M.A., Silva L.R. (2016). Mathematical Modeling of Drying Kinetics of Persimmon Fruits (Diospyros kaki cv. Fuyu). J. Food Process. Preserv..

[45] Yadollahinia A., Jahangiri M. (2009). Shrinkage of potato slice during drying. J. Food Eng..

[46] Mayor L., Sereno A.M. (2004). Modelling shrinkage during convective drying of food materials: A review. J. Food Eng..

[47] Ramallo L.A., Mascheroni R.H. (2012). Quality evaluation of pineapple fruit during drying process. Food Bioprod. Process..

[48] Miranda M., Vega-Gálvez A., García P., Di Scala K., Shi J., Xue S., Uribe E. (2010). Effect of temperature on structural properties of Aloe vera (Aloe barbadensis Miller) gel and Weibull distribution for modelling drying process. Food Bioprod. Process..

[49] Vega-Galvez A., Notte-Cuello E., Lemus-Mondaca R., Zura L., Miranda M. (2009). Mathematical modelling of mass transfer during rehydration process of Aloe vera (Aloe barbadensis Miller). Food Bioprod. Process..

[50] Benseddik A., Azzi A., Zidoune M.N., Khanniche R., Besombes C. (2019). Empirical and diffusion models of rehydration process of differently dried pumpkin slices. J. Saudi Soc. Agric. Sci..

[51] Chenlo F., Arufe S., Díaz D., Torres M.D., Sineiro J., Moreira R. (2017). Air-drying and rehydration characteristics of the brown seaweeds, Ascophylum nodosum and Undaria pinnatifida. J. Appl. Phycol..

[52] Krokida M., Marinos-Kouris D. (2003). Rehydration kinetics of dehydrated products. J. Food Eng..

[53] Tepe T.K., Tepe B. (2020). The comparison of drying and rehydration characteristics of intermittent-microwave and hot-air dried-apple slices. Heat Mass Transf..

[54] Aral S., Beşe A.V. (2016). Convective drying of hawthorn fruit (Crataegus spp.): Effect of experimental parameters on drying kinetics, color, shrinkage, and rehydration capacity. Food Chem..

[55] Xiao H.-W., Bai J.-W., Xie L., Sun D.-W., Gao Z.-J. (2015). Thin-layer air impingement drying enhances drying rate of American ginseng (Panax quinquefolium L.) slices with quality attributes considered. Food Bioprod. Process..

[56] Saberian H., Abbasi S., Hamidi-Esfahani Z. (2013). Effect of pasteurization and storage on bioactive components of Aloe vera gel. Nutr. Food Sci..

[57] Nielsen C.W., Rustad T., Holdt S.L. (2021). Vitamin C from Seaweed: A Review Assessing SeaWeed as Contributor to Daily Intake. Foods.

[58] Rudolph A., El-Mohamad A., McHardy C., Rauh C. (2021). Concentrating Model Solutions and Fruit Juices Using CO2 Hydrate Technology and Its Quantitative Effect on Phenols, Carotenoids, Vitamin C and Betanin. Foods.

[59] Nemati Z., Alirezalu K., Besharati M., Amirdahri S., Franco D., Lorenzo J.M. (2020). Improving the Quality Characteristics and Shelf Life of Meat and Growth Performance in Goose Fed Diets Supplemented with Vitamin E. Foods.

[60] Gómez-Limia L., Sanmartín N.M., Carballo J.M., Domínguez R., Lorenzo J., Martínez S. (2021). Oxidative Stability and Antioxidant Activity in Canned Eels: Effect of Processing and Filling Medium. Foods.

[61] Ouyang M., Cao S., Huang Y., Wang Y. (2021). Phenolics and ascorbic acid in pumpkin (Cucurbita maxima) slices: Effects of hot air drying and degradation kinetics. J. Food Meas. Charact..

[62] Kurozawa L.E., Terng I., Hubinger M.D., Park K.J. (2014). Ascorbic acid degradation of papaya during drying: Effect of process conditions and glass transition phenomenon. J. Food Eng..

[63] Jha A.K., Sit N. (2020). Drying characteristics and kinetics of colour change and degradation of phytocomponents and antioxidant activity during convective drying of deseeded Terminalia chebula fruit. J. Food Meas. Charact..

[64] Leiton-Ramírez Y.M., Ayala-Aponte A., Ochoa-Martínez C.I. (2020). Physicochemical Properties of Guava Snacks as Affected by Drying Technology. Processes.

[65] Kolla M.C., Laya A., Bayang J.P., Koubala B.B. (2021). Effect of different drying methods and storage conditions on physical, nutritional, bioactive compounds and antioxidant properties of doum (Hyphaene thebaica) fruits. Heliyon.

